# Rapid Determination of Selected PFAS in Textiles Entering the Waste Stream

**DOI:** 10.3390/toxics11010055

**Published:** 2023-01-06

**Authors:** Daniel Simon Drage, Martin Sharkey, Harald Berresheim, Marie Coggins, Stuart Harrad

**Affiliations:** 1 School of Geography, Earth & Environmental Sciences, University of Birmingham, Birmingham B15 2TT, UK; 2 Queensland Alliance for Environmental Health Sciences (QAEHS), The University of Queensland, 20 Cornwall Street, Woolloongabba, QLD 4103, Australia; 3 School of Natural Sciences & Ryan Institute, University of Galway, H91TK33 Galway, Ireland

**Keywords:** perfluoroalkyl substances, PFAS, waste management, polymers, LC-TOF/MS, persistent organic pollutant

## Abstract

Due to new European legislation, products entering the waste stream containing some perfluoro alkyl substances (PFAS) are subject to “low persistent organic pollutant concentration limits”. Concentrations of restricted PFAS must be below this limit for them to be legally recycled or disposed of. A rapid extraction and clean-up method was developed for the determination of 21 PFAS in various polymers used in soft furnishings and upholstery. The optimised method used vortexing and ultrasonication in methanol (0.1% NH_4_OH), followed by a dilution and syringe filter clean-up step. PFAS were subsequently determined via UPLC-TripleTOF/MS. Good recoveries (80–120%) of target analytes were obtained with tall and narrow chromatogram peaks. The method was validated using control matrix samples spiked with target analytes. Repeated measurements of concentrations of target compounds showed good agreement with the spiked concentrations demonstrating good accuracy and precision. The resultant extracts provided low noise levels resulting in low limits of quantification ranging from 0.1 to 0.4 mg/kg. The developed method was applied successfully to real consumer products and it provided various advantages over traditional methods, including a substantially reduced analysis time, consumables and solvent consumption, and a high sample throughput which is critical to comply with implemented and proposed legislation.

## 1. Introduction

Per- and polyfluoroalkyl substances (PFAS) are a class of thousands of chemical substances. These chemicals have been produced since the 1950s and still find use in a wide variety of industrial applications and consumer products. PFAS are both chemically and thermally stable, as well as having hydrophobic and lipophobic properties, making them very useful when incorporated as surfactants in firefighting foams and coatings, as well as polymers used in textiles and food packaging materials [1,2].

Various studies have raised concerns about the toxic effects of PFAS, including adverse effects on liver function, and an association between perfluorooctane sulfonate (PFOS) and perfluorooctanoic acid (PFOA) and birth weight [3] reduced antibody response to vaccination against diphtheria and tetanus [4,5] and Haemophilus influenza type b [6]. Other negative health effects resulting from PFAS exposure include endocrine disruption, e.g., thyroid toxicity and some cancers [7,8], kidney and testicular cancers [7]. 

The strong C-F bond means that PFASs are resistant to thermal, chemical, and biological degradation [9] and are capable of bioaccumulation and long-range environmental transport, exemplified by their detection in the Arctic [10]. As a result, currently, two PFAS are listed under the Stockholm Convention on POPs. PFOS, including its salts and perfluorooctane sulfonyl fluoride (PFOSF), are listed in Annex B (Restriction); while PFOA, its salts and PFOA-related compounds (including precursor compounds) are listed in Annex A (Elimination) [11]. Moreover, following the recommendation of the POPs Review Committee [12], the 10th meeting of the Conference of the Parties to the Stockholm Convention in June 2022 listed perfluorohexane sulfonic acid (PFHxS), its salts, and PFHxS-related compounds in Annex A to the Convention, setting them for elimination, with no exemptions. The POPs review committee (POPRC) is also currently considering listing under the Stockholm Convention C_9_–C_21_ analogues of PFOA [13]. In addition, perfluorobutane sulfonate (PFBS), PFHxS, PFOA, perfluorononanoic acid (PFNA), and perfluorodecanoic acid (PFDA) are listed under REACH (Regulation on Registration, Evaluation, Authorisation and Restriction of Chemicals) as substances of very high concern recommended for restriction [14].

Despite the known health effects and proposed restrictions, many items, and products containing PFAS remain in use. Moreover, in view of the turnover times of such articles, it is further apparent that there is a growing inventory of materials containing restricted chemicals that have or will shortly be entering the waste stream. To illustrate, a recent study estimated that 2651 t of end-of-life vehicle foams/fabrics and 20,949 t of soft furnishing foams/fabrics were generated in Ireland annually [15]. In its document “Manifesto for a Resource Efficient Europe” [16], the EU in keeping with other jurisdictions recognised that it has no choice but to transition to a resource-efficient and ultimately regenerative circular economy. An alternative to a traditional linear economy, a circular economy is one in which resources are kept in use for as long as possible. The maximum value is extracted from resources whilst in use, with products and materials recovered and regenerated at the end of each service life. A potential obstacle to this is the presence of PFAS in plastic components of waste such as fabrics, foams and other soft furnishings. Evidence exists that uncontrolled recycling of polymers can lead to the unintentional presence of organic contaminants, such as halogenated flame retardants in articles where their presence is not required, including food contact materials, children’s toys, and polystyrene packaging [17,18,19,20,21,22,23]. For many banned chemicals the EU has implemented Low POP Concentration Limit (LPCL) values, which forbid the recycling of waste polymers containing such chemicals at concentrations exceeding a specified value. With respect to PFAS, the EU specifies an LPCL value for PFOS of 50 mg/kg [24], while they have also recently proposed limits of 1 mg/kg for each PFOA and PFHxS [25]. Currently, there are no LPCL values for other PFAS in the waste specified. However, with the growing concern over their toxic effects and an increasing level of detection of other PFAS, it is likely that additional LPCLs or a combined LPCL for total PFAS content will be introduced. With this in mind, combined with the large volumes of waste that exist globally, it is essential that a rapid method is made available for the determination of PFAS levels in waste polymers. Current methods exist, however these often involve several sample preparation steps including solid phase extraction (SPE) and sample concentration (Table 1) [26,27,28,29,30]. This can be time-consuming and expensive requiring large amounts of organic solvent and sample preparation materials, which is not suitable when considering the high throughput of samples that would be required to deal with the current volumes of waste produced. Therefore, the aim of this study is to: (i) develop a simple, sensitive, rapid and high throughput method for testing waste polymers used in soft furnishings (including textiles, furniture foams, carpets and curtains) for compliance with LPCLs and similar legislation for the most frequently detected PFAS in these articles; (ii) validate the methods using matrix spiked reference materials; and (iii) apply the validated method to the analysis of real waste samples. 

## 2. Materials and Methods

### 2.1. Chemicals and Reagents

Individual native PFAS (PFBS, PFHxS, PFHpS, PFOS, PFNS, PFDS, PFPeA, PFHxA, PFHpA, PFOA, PFNA, PFDA, PFUdA, PFDoA, PFTrDA, PFTeDA, FOSA, MeFOSA, EtFOSA, MeFOSE and EtFOSE) and labelled PFAS (MPFBS, MPHFxS, M8PFOS, M8PFOA, MPFPeA, MPFHxA, MPFHpA, MPFNA, MPFDA, MPFUdA, MPFDoA, MPFTeDA, M8FOSA, d-MeFOSA, d_7_-MeFOSE) standards were purchased from Wellington Laboratories (Guelph, ON, Canada). All other chemicals and solvents (HPLC Grade dichloromethane (DCM), LC Optima Grade methanol and LC Optima Grade water, ammonia solution) were purchased from Fisher Scientific (Loughborough, UK). 

### 2.2. Sample Collection

Waste carpet, curtain and end-of-life vehicle upholstery samples were collected from waste sorting facilities across Ireland during 2015 and 2016 as part of a separate study [15]. Five samples of each matrix from these three waste categories (totalling 15 samples) were selected at random for the purposes of this study.

### 2.3. Sample Extraction and Preparation

Small sections (approximately 10 cm × 10 cm) were cut into small strips using methanol-rinsed scissors. Samples were then accurately weighed (100 mg) into a clean 15 mL glass tube and spiked with 50 ng of each internal standard. Five millilitres of methanol (0.1% NH_4_OH) was added to the tubes (step 1), which were vortexed for two minutes (step 2), followed by 20 min of sonication at 20 ℃ (step 3). Steps 2 and 3 were then repeated two further times (totalling 6 min of vortexing and 60 min of sonication). The tubes were centrifuged at 2500 RPM (1000× *g*) for 5 min. One millilitre of the extract was passed through a 0.21 μm PES syringe filter and into a 1.5 mL amber vial and stored at −20 ℃ until analysis. 

### 2.4. Chemical Analysis

Selected PFAS (PFBS, PFHxS, PFHpS, PFOS, PFNS, PFDS, PFPeA, PFHxA, PFHpA, PFOA, PFNA, PFDA, PFUdA, PFDoA, PFTrDA, PFTeDA, FOSA, MeFOSA, EtFOSA, MeFOSE and EtFOSE) were determined using a Sciex Exion UPLC, coupled to a Sciex 5600+ triple TOF MS. Ten microlitres of the sample extract were injected onto a Raptor C18 column (1.8 µm particle size, 50 mm length, 2.1 mm internal diameter, Restek). At a flow rate of 0.4 mL/min, a mobile phase gradient was ramped from 80% Mobile Phase A (5 mM ammonium formate in water), 20% mobile phase B (5 mM ammonium formate in MeOH) to 95% mobile phase B over 6 min. This was held for 0.5 min before equilibrating back to 20% mobile phase B for 1.5 min. The triple TOFMS was equipped with a Turbo V source which was operated in negative mode using electrospray ionisation at a voltage of −4500 V. The curtain gas was set at 25 psi, whilst the nebuliser gas (source gas 1) was set at 25 psi and the drying gas (source gas 2) at 35 psi. The collision-activated dissociation (CAD) gas was set to medium and the temperature was 450 °C. The TOFMS was operated in full scan mode scanning from 100–1000 Da. Manual mass calibration was performed on the instrument prior to the injection of each batch of samples using a Sciex calibrant delivery system (CDS) to ensure a starting mass accuracy of <1 ppm. An automated calibration was also performed after every 10 injections to ensure ongoing mass accuracy throughout each run. Concentrations of selected PFAS were measured using isotope dilution, and a full list of compounds with their respective monitoring ions and internal standards is presented in Table S1 (supporting information (SI)).

### 2.5. Quality Assurance/Quality Control

A reagent blank consisting of 100 mg of anhydrous sodium sulphate was analysed with every batch of 9 samples. “Control” samples were created using textiles that contain no PFASs and were also analysed throughout the study. None of the target compounds were found above the limits of detection in the blanks. Therefore, results were not corrected for blank residues and method limits of detection (LOD) and quantification (LOQ) were estimated based on a signal-to-noise ratio (S/N) of 3:1 and 10:1, respectively. LODs and LOQs for each target PFAS are provided in Table S2 (SI).

For a given peak to be identified as a target pollutant in a sample, the following criteria needed to be met:

(1) The S/N must exceed 3:1;
(2) The m/z value must be within 50 ppm of the accurate mass determined for each analyte;
(3) The relative retention time (RRT) of the peak in the sample must be within ±0.2% of the average value determined for the same congener in the 2 calibration sets run before and after that sample batch.

In the absence of appropriate certified reference material, a matrix spike was analysed for every 20th sample and was required to be within 80–120% of the spiked concentration. 

## 3. Results and Discussion

### 3.1. Optimisation of Method Parameters

Several initial experiments were conducted during method development designed to optimise sample preparation parameters with the aim of improving extraction efficiency for all target compounds, whilst minimising sample preparation time and chromatographic interferences. These experiments were classified into two main categories:

#### 3.1.1. Optimisation of Extraction

**Figure 1:**
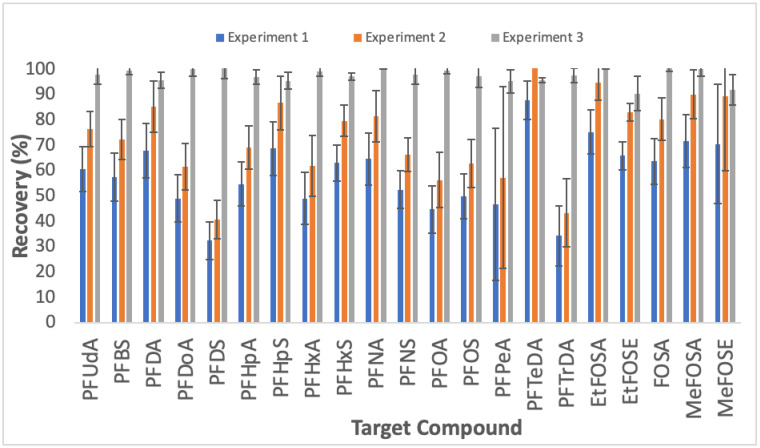
Recoveries of PFAS (all 1 mg/kg) with varying number of extraction cycles (Experiment 1 = 1 cycle; Experiment 2 = 2 cycles; Experiment 3 = 3 cycles).

The number of previous studies determining PFAS is low; however, one previous study suggests sonication of the sample overnight [26], while another suggests sonication for only 30 min [29]. In our method, one full extraction cycle consisted of 2 min of vortexing followed by 20 min of sonication. We tested the effect on the recovery of repeating the extraction cycle by fortifying samples with native standards (at 1 mg/kg concentration) overnight, and then performing the following experiments: (i) one extraction cycle (n = 5); (ii) two extraction cycles (n = 5); and (iii) three extraction cycles (n = 5). Experiments (i) and (ii) produced final recoveries that were below the acceptable limits with average recoveries of 59% (range = 33–88%) and 74% (range = 41–110%) for experiments (i) and (ii), respectively (Figure 1). Furthermore, while the addition of a second extraction cycle in an experiment (ii) increased the average recovery for all compounds, there was still considerable variability for some compounds (average = 11%; range = 3.4–36%). The addition of the third extraction step in an experiment (iii) substantially improved both extraction efficiency and variability with an average recovery of 98% (range = 91–100%) and an average RSD of 3.3% (range = 1–6.8%). It was therefore determined that three full extraction cycles were necessary.

#### 3.1.2. Effect of Extract Filtration on Chromatography

**Figure 2:**
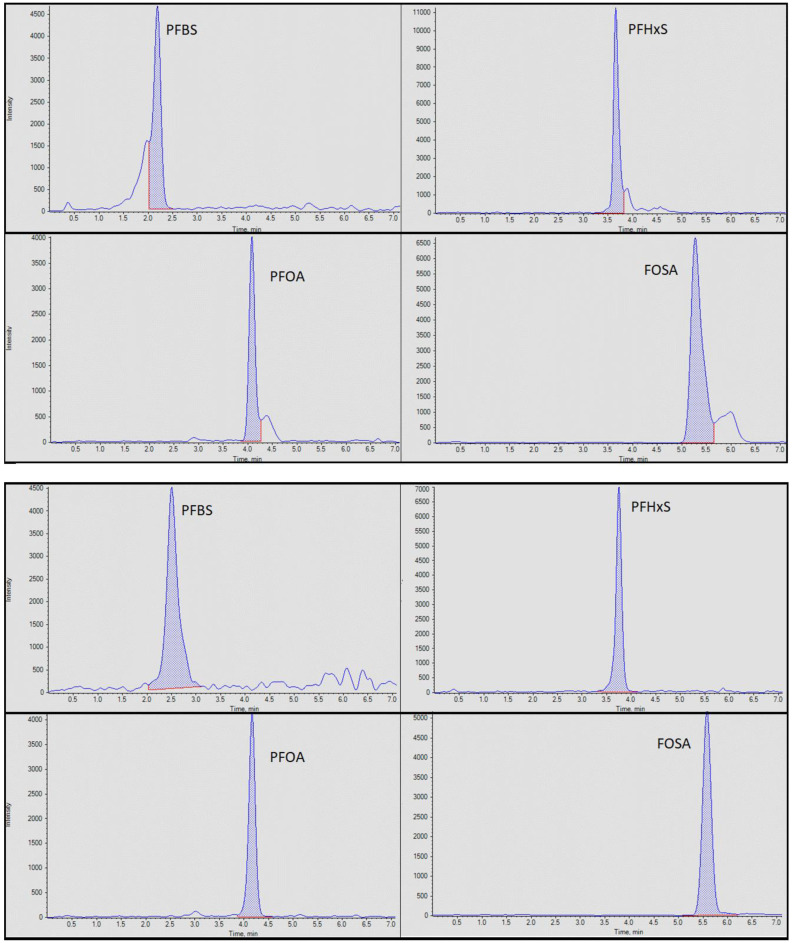
Chromatograms in test samples for PFBS, PFHxS, PFOA, and FOSA (all 1 mg/kg) before (**top**) and after (**bottom**) syringe filtration.

Of the few studies that have determined PFAS in textile samples, there have been different approaches to preparing the extract prior to chemical analysis. One study uses gravimetric filtration through qualitative filter paper [26], while another uses SPE clean-up on an ENVI-carb cartridge [30], which is regularly used in a variety of methods for the determination of PFAS in various matrices [31,32,33,34]. Both can be time-consuming, and the former involves some losses of target compounds, which can increase the limits of quantification. We investigated the effects of syringe filtration on chromatography by performing triplicate measurements of an extract before and after passing through a syringe filter. The use of a syringe filter is a rapid process that will remove any particulate matter and large molecules from the extract within a few seconds. As it is the final step of the procedure it is likely to have a negligible effect on the recoveries of our target PFAS. Figure 2 demonstrates that the use of a syringe filter clearly improved chromatography for several target PFAS compounds by removing interferences producing narrower and more symmetric peaks making identification and integration more straightforward. By eliminating shoulders from the peaks of several compounds, this step also improves the S/N ratio of our target PFAS maximising the sensitivity of the method and optimising LOQs. It was therefore decided that this was a necessary and satisfactory rapid clean-up step for this method. 

### 3.2. Method Validation

#### 3.2.1. Linearity and Range

A 1/x weighted linear regression (R^2^ > 0.995) calibration curve containing eight points was constructed successfully for each target compound (with at least 3 measurements at each concentration level) over a wide concentration range (0.5 pg/μL–200 pg/μL) using the assigned internal standards. Relative response factors (RFs) were estimated for each target compound. The relative standard deviation (RSD) of RFs for each target compound did not exceed 5%. 

#### 3.2.2. Method Accuracy and Precision

In the absence of a certified reference material (CRM), method accuracy and precision were assessed via repeated analysis of spiked matrix samples. A control textile sample containing was aliquoted and accurately weighed (100 mg) into 6 tubes. One tube was left unspiked as a control, and the remaining 5 tubes were spiked with 100 ng of target compounds (i.e., 1 mg/kg). Samples were left at <4 ℃ to fortify overnight and then subjected to the same procedures as in Section 2.3 and analysed according to the same procedures as in Section 2.4. The recoveries of each target analyte were then calculated according to the following equation:

$$Recovery\;\left(\%\right)=\frac{C_{M}}{C_{S}}\times 100$$

where *C_M_* is the measured concentration; and *C_S_* is the spiked concentration of each target analyte. The recoveries presented in Table 2 demonstrate that all measured concentrations were 80–120% of the spiked concentration levels with an RSD of <15%. The consistently high recoveries of target analytes (average = 98%, range = 91–104%) along with the low RSD between repeated measurements (average = 3.3%, range: 1–6.8%) demonstrates that this is an accurate, precise and robust method for the determination of PFAS in various textile samples. The results have demonstrated the requirement for 3 cycles of vortexing (3 × 2 min = 6 min total) and sonication (3 × 20 min = 60 min total) followed by centrifugation (5 min) and syringe filtering an aliquot of the extract. This confirms the simplicity (i.e., minimal number of steps) of the developed method and allows for a rapid and high throughput of samples for analysis.

#### 3.2.3. Sensitivity, Limits of Detection and Quantification

The method achieved consistently high recoveries of target compounds in the lower half of the calibration range for each of the studied compounds. No interference was observed in the method blanks or controls analysed alongside the samples. This combined with a low baseline (Figure 2) meant that the method achieved high sensitivity and low detection limits. Instrumental method LODs were estimated based on a 3:1 S/N ratio (Table S2). The LOQ was determined by a concentration equivalent to an S/N ratio of 10:1 in the samples (range: 0.1–0.4 mg/kg). These were considered satisfactory given that it is believed that consumer products have been treated at considerable concentrations, whilst the current regulated limits for PFOS (50 mg/kg) [24] are 500 times higher than our method LOQ, and the LPCLs for PFOA and PFHxS (1 mg/kg for each) are 10 times higher than our method LOQ. 

### 3.3. Application to Real Samples

The developed analytical method was applied to the analysis of real textile samples entering the waste stream. These comprised 15 samples (5 carpet samples, 5 curtain samples and 5 end-of-life vehicle (ELV) upholstery samples) collected from waste treatment sites across Ireland. Our analytical method displayed good performance evidenced by the high internal standard peaks along with a low baseline in the mass chromatograms (Figure S1). 

At least one individual PFAS was detected in 7 out of 15 samples with an average ∑PFAS (sum of all 21 targets PFAS in this study (Table 3)) concentration of 2.0 mg/kg (range = (<LOQ–18 mg/kg). They were detected in 2 out of 5 carpet samples at an average ∑PFAS concentration of 1.1 mg/kg (range = <LOQ–4.6 mg/kg). They were detected in 4 out of 5 curtain samples at an average concentration of 1.3 mg/kg (range = <LOQ = 4.4 mg/kg); and in 1 out of 5 ELV samples (18 mg/kg).

Samples displayed no pattern based on waste classification, with individual PFAS having low detection frequencies (DF) (average= 8.9%; range = 0–27%). The most regularly detected PFAS was FOSA, which was detected in three curtain samples and one carpet sample at an average concentration of 0.2 mg/kg. The highest ∑PFAS concentration measured was in “ELV 2” (an upholstery sample from a car), which contained 18.4 mg/kg, 92% of which was PFHxA. PFHxA was also the dominant PFAS in the second most concentrated sample making up 57% of the ∑PFAS content in “Carpet 5” (4.6 mg/kg). 

Interestingly, only one sample (Curtain 2) contained detectable levels of PFOS (0.7 mg/kg), which is >50 times lower than the current LPCL in waste [35]. While the total number of samples analysed is small, this indicates that Ireland does not have an overwhelming volume of waste requiring special treatment due to its PFOS content. This coincides with recent studies in Irish waste and other human exposure studies which all found low levels of PFOS compared to other European locations [36,37,38]. Two other PFAS that are listed as POPs under the Stockholm Convention are PFOA and PFHxS [39,40]. While their LPCLs are 50 times lower than that of PFOS, PFOA was not detected in any samples, and PFHxS was only detected in one sample at a low concentration of 0.15 mg/kg (>6 times below the recommended LPCL). Further sampling and analysis are required to determine the extent of waste requiring special treatment in Ireland. 

## 4. Summary

A rapid, simple and sensitive method was developed for the extraction and determination of PFAS in textiles by LC-TOF/MS. The method involved a combination of vortexing and ultrasonication followed by syringe filtering to remove interfering particulates and macromolecules from the extract. The method was validated using matrix spikes and displayed good accuracy and precision. Application of the validated method to a limited number of real samples of textiles entering the waste stream revealed some interesting results. With legislation already prescribing an LPCL for PFOS in products entering the waste stream and recommended for PFOA and PFHxS, a rapid method for their determination in waste textiles is pertinent as items above these limits cannot be recycled. The developed method provided advantages over previous methods including reduced solvent consumption, shorter analysis time and enhanced recovery of target analytes, allowing for high sample throughput that will expedite future monitoring of compliance with LPCLs.

## Figures and Tables

**Table 1:** Existing analytical methodologies for determination of PFAS and their disadvantages against testing LPCL compliance in a high throughput environment.

Study	Disadvantage
**Peaslee et al. 2020** [26]	- Requires a relatively large sample (250 mg); - Requires a relatively large volume of solvent (15 mL) - Long extraction time (overnight sonication)
**Janousek et al. (2019)** [27]	- Requires large sample (1 g) - Requires a large volume of solvent (20 mL) - Relatively long processing time (2-h extraction, “several hours” cooling, and sample fractionation/concentration - Observed matrix effects
**Xia et al. (2022)** [28]	- Long sample processing time (multiple extraction steps, sample concentration and dispersive SPE clean-up step)
**Rewerts et al. (2018)** [29]	- Targets primarily fluorotelomer alcohols, and does not target the PFAS addressed in this study - Method is designed for a GC/MS application
**Verstergen et al. (2015)** [30]	- Long sample processing time (requires 2 extract concentration steps and an SPE clean-up step)

**Table 2:** Measured concentrations (mg/kg) and recoveries (%) of target analytes in matrix spiked samples.

		Measured Concentration						Recovery (%)						
Compound	Spiked Conc.	Control	Val_001	Val_002	Val_003	Val_004	Val_005	Val_001	Val_002	Val_003	Val_004	Val_005	RSD	Average
PFUdA	1 mg/kg	<1.0	0.98	1.02	1.02	0.96	0.93	98.0	98.0	102.0	101.6	96.4	2.8	99.5
PFBS	1 mg/kg	<1.0	0.98	0.99	1.02	0.98	1.01	98.5	98.5	99.3	101.6	98.0	1.6	99.3
PFDA	1 mg/kg	<1.0	0.97	0.95	0.93	0.93	1.00	97.4	97.4	94.6	93.5	92.7	2.2	94.5
PFDoA	1 mg/kg	<1.0	0.98	1.05	0.98	0.98	1.02	98.2	98.2	104.6	98.2	98.4	3.2	99.8
PFDS	1 mg/kg	<1.0	0.97	0.97	1.05	1.00	1.06	97.0	97.0	96.6	105.4	99.9	4.0	99.7
PFHpA	1 mg/kg	<1.0	0.95	0.99	0.94	1.01	0.96	94.9	94.9	98.7	94.5	101.1	3.2	97.3
PFHpS	1 mg/kg	<1.0	0.93	0.94	1.00	0.94	0.98	92.6	92.6	93.9	100.3	93.8	3.7	95.1
PFHxA	1 mg/kg	<1.0	0.99	1.00	1.00	1.01	0.96	98.7	98.7	99.8	100.3	101.1	1.0	99.9
PFHxS	1 mg/kg	<1.0	0.95	0.98	0.99	0.96	0.98	95.2	95.2	97.9	98.7	96.1	1.6	96.9
PFNA	1 mg/kg	<1.0	1.03	1.03	0.99	1.07	1.03	102.9	102.9	102.6	99.5	107.1	3.0	103.0
PFNS	1 mg/kg	<1.0	0.95	0.98	1.03	0.94	1.00	94.7	94.7	98.5	103.0	94.1	4.2	97.5
PFOA	1 mg/kg	<1.0	1.00	1.01	0.99	0.98	1.00	100.1	100.1	100.7	98.9	97.7	1.3	99.3
PFOS	1 mg/kg	<1.0	0.97	0.95	1.01	0.92	1.03	96.6	96.6	95.0	100.9	91.8	4.0	96.0
PFPeA	1 mg/kg	<1.0	0.93	0.90	0.96	0.95	1.03	92.9	92.9	90.4	95.8	95.1	2.6	93.5
PFTeDA	1 mg/kg	<1.0	0.95	0.96	0.95	0.98	0.95	95.2	95.2	95.7	95.4	97.6	1.2	96.0
PFTrDA	1 mg/kg	<1.0	0.96	1.02	0.95	0.99	0.96	96.0	96.0	101.9	95.2	98.9	3.1	98.0
EtFOSA	1 mg/kg	<1.0	1.00	1.01	1.05	1.07	1.06	99.5	99.5	100.8	104.8	107.3	3.5	103.1
EtFOSE	1 mg/kg	<1.0	0.99	0.81	0.89	0.88	0.95	79.3	99.1	81.4	88.9	88.1	8.1	89.4
FOSA	1 mg/kg	<1.0	1.04	0.98	1.05	1.00	1.04	103.9	103.9	98.2	104.6	100.1	3.0	101.7
MeFOSA	1 mg/kg	<1.0	0.99	0.99	1.02	0.97	1.04	98.5	98.5	98.7	102.2	97.3	2.1	99.2
MeFOSE	1 mg/kg	<1.0	1.00	0.90	0.91	0.84	0.95	100.2	100.2	90.2	90.7	83.7	7.4	91.2

**Table 3:** Concentrations (mg/kg) of PFAS in textiles entering the waste stream in Ireland.

PFAS Compound	Carpet 1	Carpet 2	Carpet 3	Carpet 4	Carpet 5	Curtains 1	Curtains 2	Curtains 3	Curtains 4	Curtains 5	ELV 1	ELV 2	ELV 3	ELV 4	ELV 5
PFBS	<0.1	<0.1	0.18	<0.1	<0.1	<0.1	0.11	<0.1	<0.1	0.12	<0.1	<0.1	<0.1	<0.1	<0.1
PFHxS	<0.1	<0.1	<0.1	<0.1	<0.1	<0.1	<0.1	<0.1	<0.1	<0.1	<0.1	0.15	<0.1	<0.1	<0.1
PFHpS	<0.1	<0.1	<0.1	<0.1	<0.1	<0.1	0.14	<0.1	<0.1	<0.1	<0.1	<0.1	<0.1	<0.1	<0.1
PFOS	<0.1	<0.1	<0.1	<0.1	<0.1	<0.1	0.7	<0.1	0.87	0.15	<0.1	<0.1	<0.1	<0.1	<0.1
PFNS	<0.1	<0.1	<0.1	<0.1	<0.1	<0.1	<0.1	<0.1	<0.1	<0.1	<0.1	0.15	<0.1	<0.1	<0.1
PFDS	<0.1	<0.1	0.14	<0.1	0.16	<0.1	<0.1	<0.1	<0.1	<0.1	<0.1	0.55	<0.1	<0.1	<0.1
PFPeA	<0.4	<0.4	<0.4	<0.4	<0.4	<0.4	<0.4	<0.4	<0.4	<0.4	<0.4	<0.4	<0.4	<0.4	<0.4
PFHxA	<0.1	<0.1	<0.1	<0.1	2.6	<0.1	<0.1	<0.1	<0.1	<0.1	<0.1	17	<0.1	<0.1	<0.1
PFHpA	<0.1	<0.1	<0.1	<0.1	<0.1	<0.1	0.12	<0.1	0.13	<0.1	<0.1	<0.1	<0.1	<0.1	<0.1
PFOA	<0.1	<0.1	<0.1	<0.1	<0.1	<0.1	<0.1	<0.1	<0.1	<0.1	<0.1	<0.1	<0.1	<0.1	<0.1
PFNA	<0.1	<0.1	<0.1	<0.1	<0.1	<0.1	<0.1	<0.1	<0.1	<0.1	<0.1	<0.1	<0.1	<0.1	<0.1
PFDA	<0.1	<0.1	<0.1	<0.1	0.53	<0.1	<0.1	<0.1	<0.1	<0.1	<0.1	<0.1	<0.1	<0.1	<0.1
PFUdA	<0.2	<0.2	0.17	<0.2	0.2	0.1	<0.2	<0.2	<0.2	<0.2	<0.2	<0.2	<0.2	<0.2	<0.2
PFDoA	<0.2	<0.2	<0.2	<0.2	1.1	<0.2	<0.2	<0.2	<0.2	<0.2	<0.2	0.52	<0.2	<0.2	<0.2
PFTrDA	<0.2	<0.2	<0.2	<0.2	<0.2	<0.2	<0.2	<0.2	<0.2	<0.2	<0.2	<0.2	<0.2	<0.2	<0.2
PFTeDA	<0.2	<0.2	<0.2	<0.2	<0.2	<0.2	<0.2	<0.2	<0.2	<0.2	<0.2	<0.2	<0.2	<0.2	<0.2
FOSA	<0.1	<0.1	0.2	<0.1	<0.1	<0.1	0.16	<0.1	0.28	0.14	<0.1	<0.1	<0.1	<0.1	<0.1
MeFOSA	<0.2	<0.2	<0.2	<0.2	<0.2	<0.2	<0.2	<0.2	<0.2	<0.2	<0.2	<0.2	<0.2	<0.2	<0.2
EtFOSA	<0.2	<0.2	<0.2	<0.2	<0.2	<0.2	<0.2	<0.2	<0.2	<0.2	<0.2	<0.2	<0.2	<0.2	<0.2
MeFOSE	<0.2	<0.2	<0.2	<0.2	<0.2	0.15	<0.2	<0.2	1.7	<0.2	<0.2	<0.2	<0.2	<0.2	<0.2
EtFOSE	<0.2	<0.2	<0.2	<0.2	<0.2	<0.2	0.3	<0.2	1.4	<0.2	<0.2	<0.2	<0.2	<0.2	<0.2
∑PFAS	<LOQ	<LOQ	0.69	<LOQ	4.59	0.25	1.6	<LOQ	4.38	0.41	<LOQ	18.37	<LOQ	<LOQ	<LOQ

## Data Availability

Not applicable.

## Supplementary Materials

The following supporting information can be downloaded at: https://www.mdpi.com/article/10.3390/toxics11010055/s1, Figure S1: Internal standard chromatograms from a typical sample; Table S1: monitoring ions (+/− 50 ppm) of PFAS targeted in this study and their respective internal standards; Table S2: Method LODs (pg/μL) and LOQs (mg/kg) for all target PFAS in this study.
