# Supplementary material for: Rapid Determination of Selected PFAS in Textiles Entering the Waste Stream
Source: Toxics. 2023 Jan 6;11(1):55. doi: 10.3390/toxics11010055 (PMC9860823; doi:10.3390/toxics11010055)
Supplement: Supplementary file 1 [file toxics-11-00055-s001.zip › toxics-2084119-supplementary.pdf]

Table S1. monitoring ions (+/- 50 ppm) of PFAS targeted in this study and their respective internal standards.

Compound	m/z	Internal Standard	m/z
**PFBS**	298.94299	**MPFBS**	301.95306
**PFHxS**	398.93660	**MPFHxS**	402.94509
**PFHpS**	448.93341	**M8PFOS**	506.95706
**PFOS**	498.93022	**M8PFOS**	506.95706
**PFNS**	548.92702	**M8PFOS**	506.95706
**PFDS**	598.92383	**M8PFOS**	506.95706
**PFPeA**	218.98618	**MPFPeA**	222.99959
**PFHxA**	312.97281	**M5PFHxA**	317.98959
**PFHpA**	318.97979	**MPFHpA**	322.99321
**PFOA**	412.96642	**M8PFOA**	420.99326
**PFNA**	462.96323	**M9PFNA**	471.99343
**PFDA**	468.97021	**MPFDA**	474.99034
**PFUdA**	562.95684	**MPFUdA**	569.98033
**PFDoA**	612.95365	**MPFDoA**	614.96036
**PFTrDA**	662.95046	**MPFTeDA**	670.96414
**PFTeDA**	712.94726	**MPFTeDA**	670.96414
**FOSA**	496.93837	**M8FOSA**	504.96521
**MeFOSA**	511.96185	**d3-MeFOSA**	514.98068
**MeFOSE**	600.98572	**d7-MeFOSE**	608.09658
**EtFOSA**	525.9775	**d3-MeFOSA**	514.98068
**EtFOSE**	616.00919	**d7-MeFOSE**	608.09658

Table S2. Method LODs (pg/μL) and LOQs (mg/kg) for all target PFAS in this study

Compound	LOD (pg/μL)	LOQ (mg/kg)
**PFUdA**	1.2	0.2
**PFBS**	0.6	0.1
**PFDA**	0.6	0.1
**PFDoA**	1.2	0.2
**PFDS**	0.6	0.1
**PFHpA**	0.6	0.1
**PFHpS**	0.6	0.1
**PFHxA**	0.6	0.1
**PFHxS**	0.6	0.1
**PFNA**	0.6	0.1
**PFNS**	0.6	0.1
**PFOA**	0.6	0.1
**PFOS**	0.6	0.1
**PFPeA**	2.4	0.4
**PFTeDA**	1.2	0.2
**PFTrDA**	1.2	0.2
**EtFOSA**	1.2	0.2
**EtFOSE**	1.2	0.2
**FOSA**	0.6	0.1
**MeFOSA**	1.2	0.2
**MeFOSE**	1.2	0.2

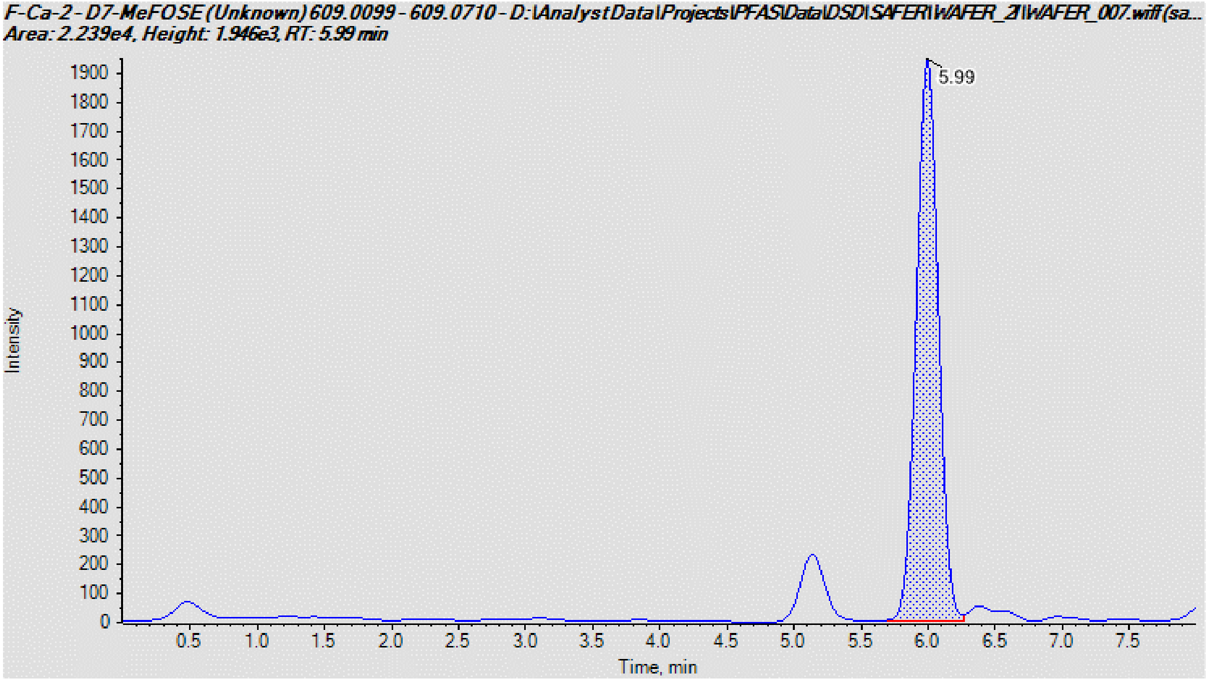

F-Ca-2 - D7-MeFOSE (Unknown) 609.0099 - 609.0710 - D:\AnalystData\Projects\PFAS\Data\DSD\SAFER\WAFER_2\WAFER_007.wiff (sa...
Area: 2.239e4, Height: 1.946e3, RT: 5.99 min
5.99
Intensity
Time, min

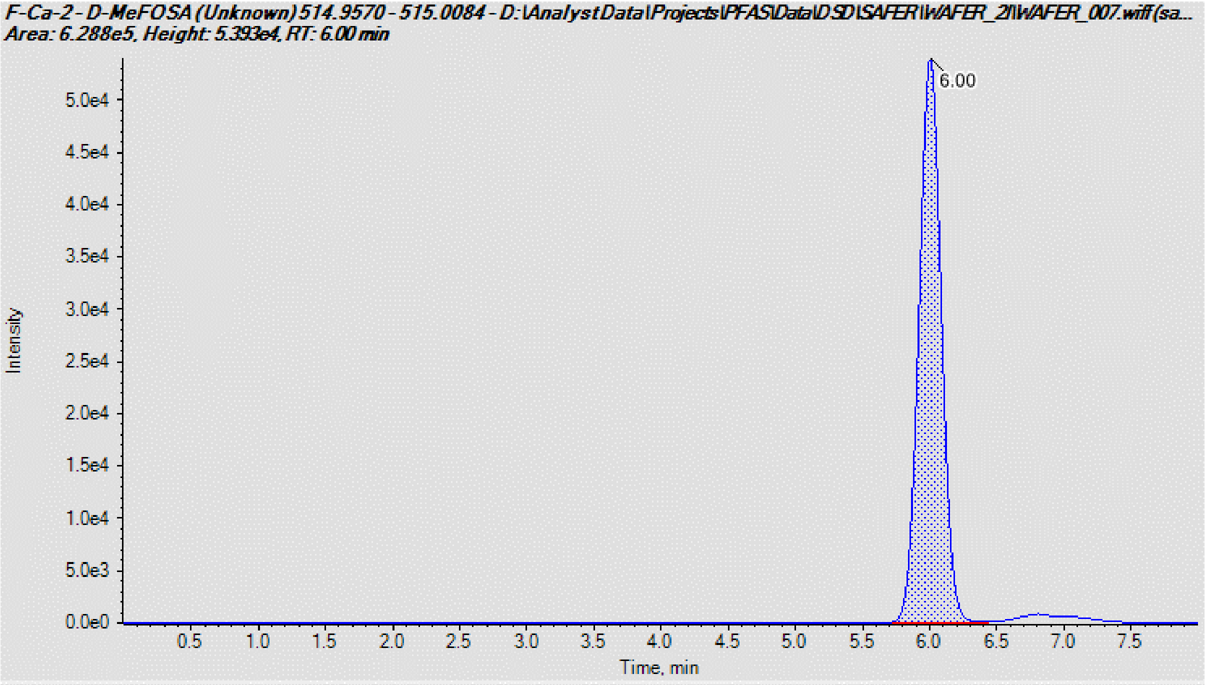

F-Ca-2 - D-MeFOSA (Unknown) 514.9570 - 515.0084 - D:\AnalystData\Projects\PFAS\Data\DSD\SAFER\WAFER_2\WAFER_007.wiff (sa...
Area: 6.288e5, Height: 5.393e4, RT: 6.00 min
6.00
Intensity
Time, min

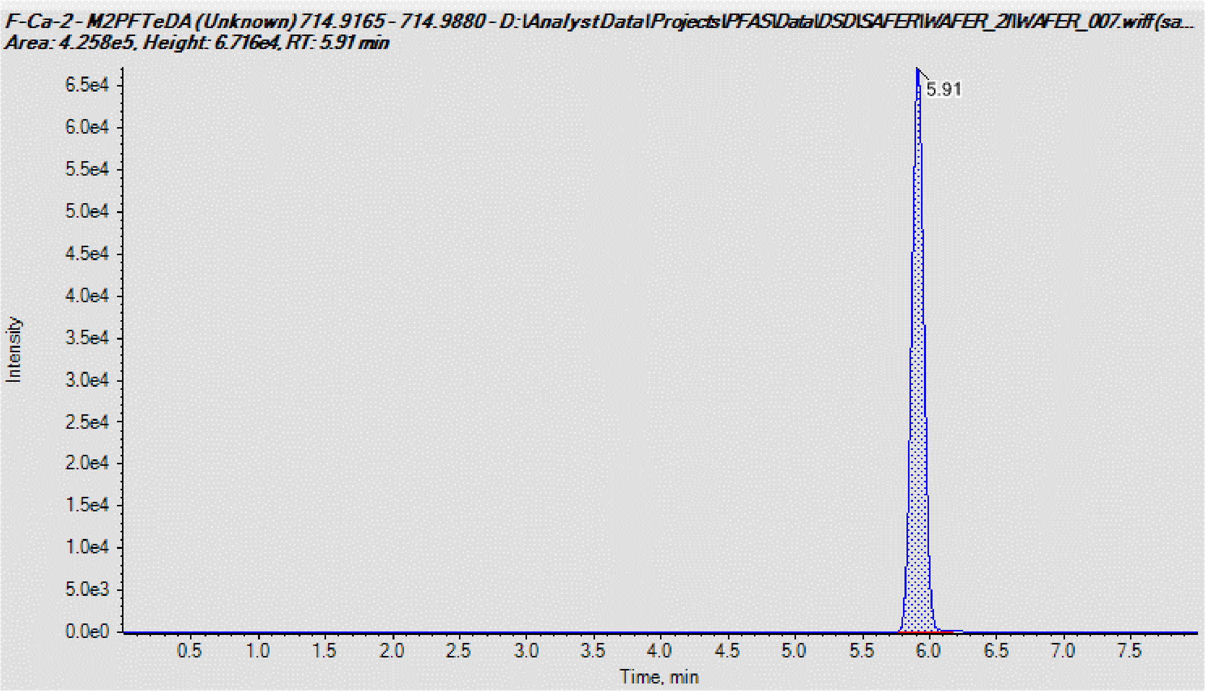
F-Ca-2 - M2PFTeDA (Unknown) 714.9165 - 714.9880 - D:\AnalystData\Projects\PFAS\Data\DSD\SAFER\WAFER_2\\WAFER_007.wiff (sa...
Area: 4.258e5, Height: 6.716e4, RT: 5.91 min
Intensity
6.5e4
6.0e4
5.5e4
5.0e4
4.5e4
4.0e4
3.5e4
3.0e4
2.5e4
2.0e4
1.5e4
1.0e4
5.0e3
0.0e0
5.91
0.5
1.0
1.5
2.0
2.5
3.0
3.5
4.0
4.5
5.0
5.5
6.0
6.5
7.0
7.5
Time, min

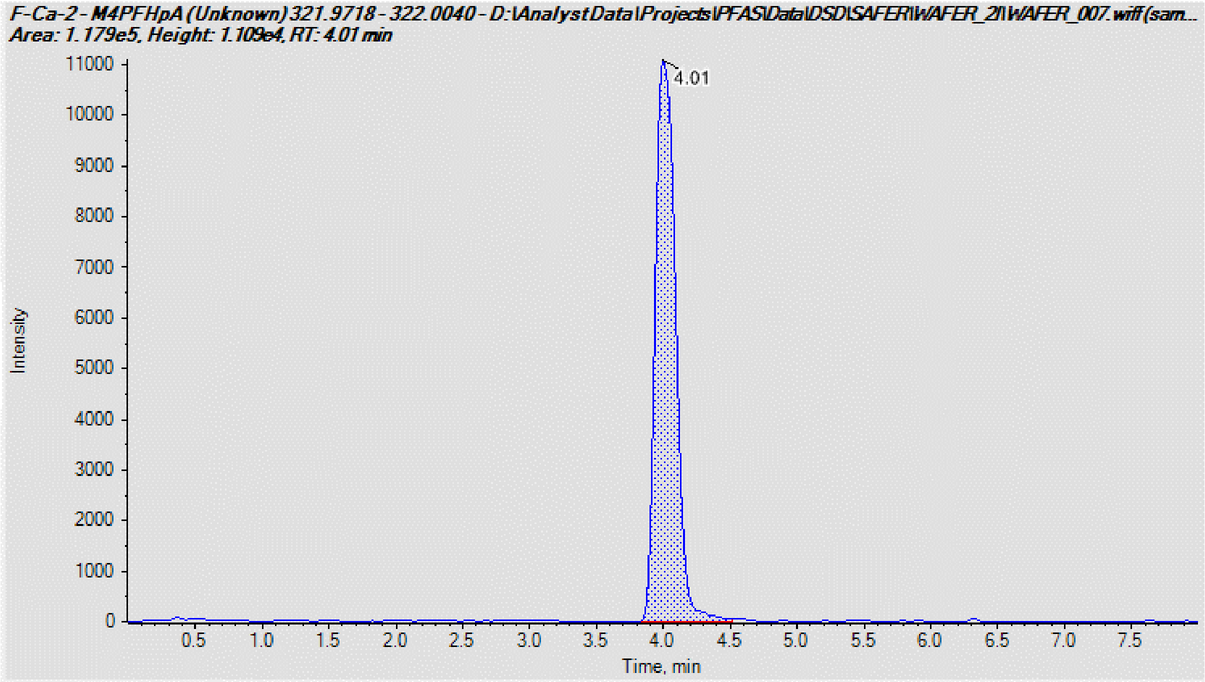
F-Ca-2 - M4PFHpA (Unknown) 321.9718 - 322.0040 - D:\AnalystData\Projects\PFAS\Data\DSD\SAFER\WAFER_2\\WAFER_007.wiff (sam...
Area: 1.179e5, Height: 1.109e4, RT: 4.01 min
Intensity
11000
10000
9000
8000
7000
6000
5000
4000
3000
2000
1000
0
4.01
0.5
1.0
1.5
2.0
2.5
3.0
3.5
4.0
4.5
5.0
5.5
6.0
6.5
7.0
7.5
Time, min

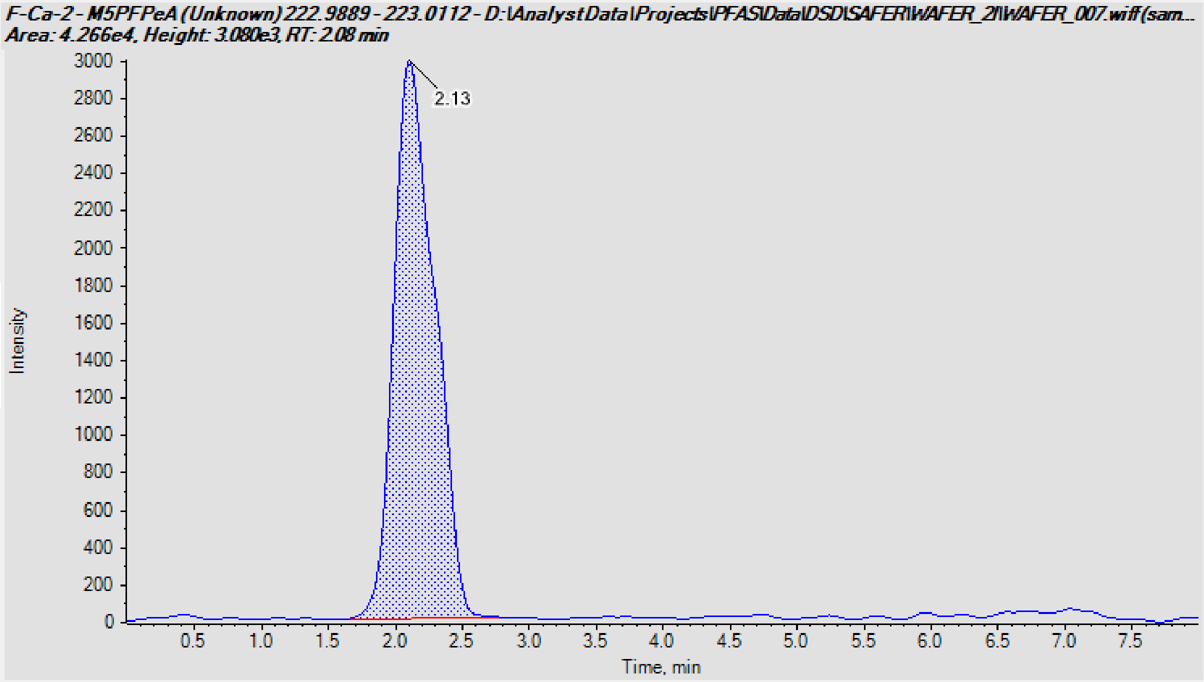
F-Ca-2 - M5PFPeA (Unknown) 222.9889 - 223.0112 - D:\AnalystData\Projects\PFAS\Data\DSD\SAFER\WAFER_2I\WAFER_007.wiff (sam...
Area: 4.266e4, Height: 3.080e3, RT: 2.08 min
2.13
Intensity
Time, min

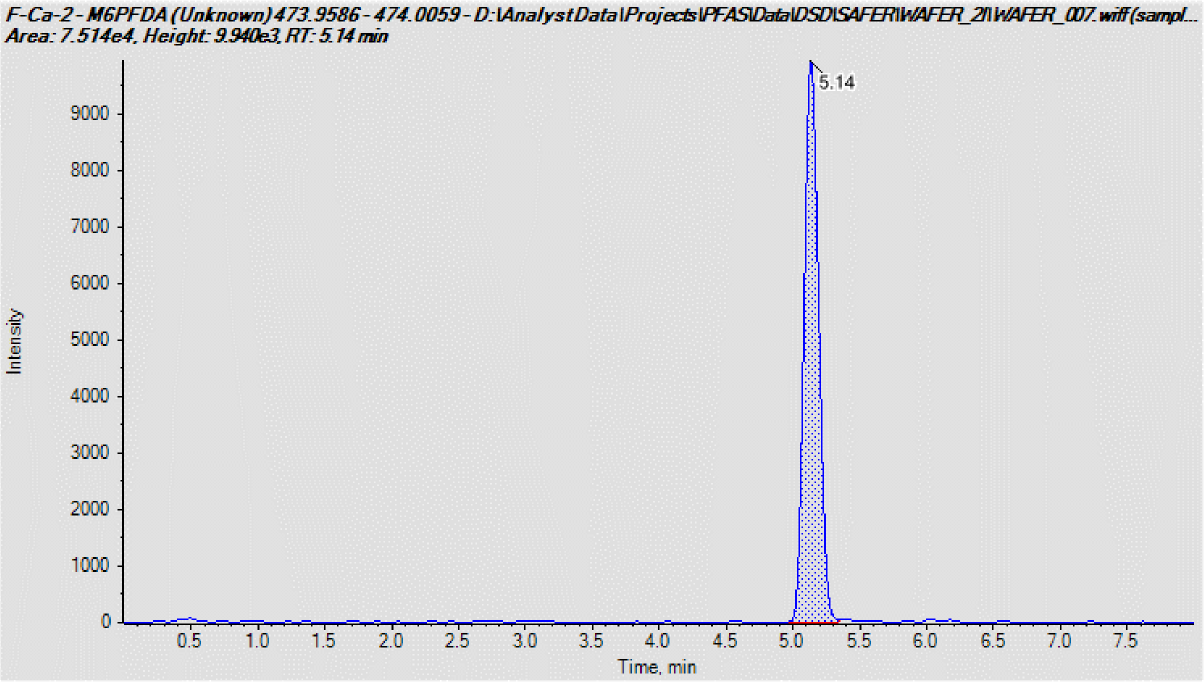
F-Ca-2 - M6PFDA (Unknown) 473.9586 - 474.0059 - D:\AnalystData\Projects\PFAS\Data\DSD\SAFER\WAFER_2I\WAFER_007.wiff (sampl...
Area: 7.514e4, Height: 9.940e3, RT: 5.14 min
5.14
Intensity
Time, min

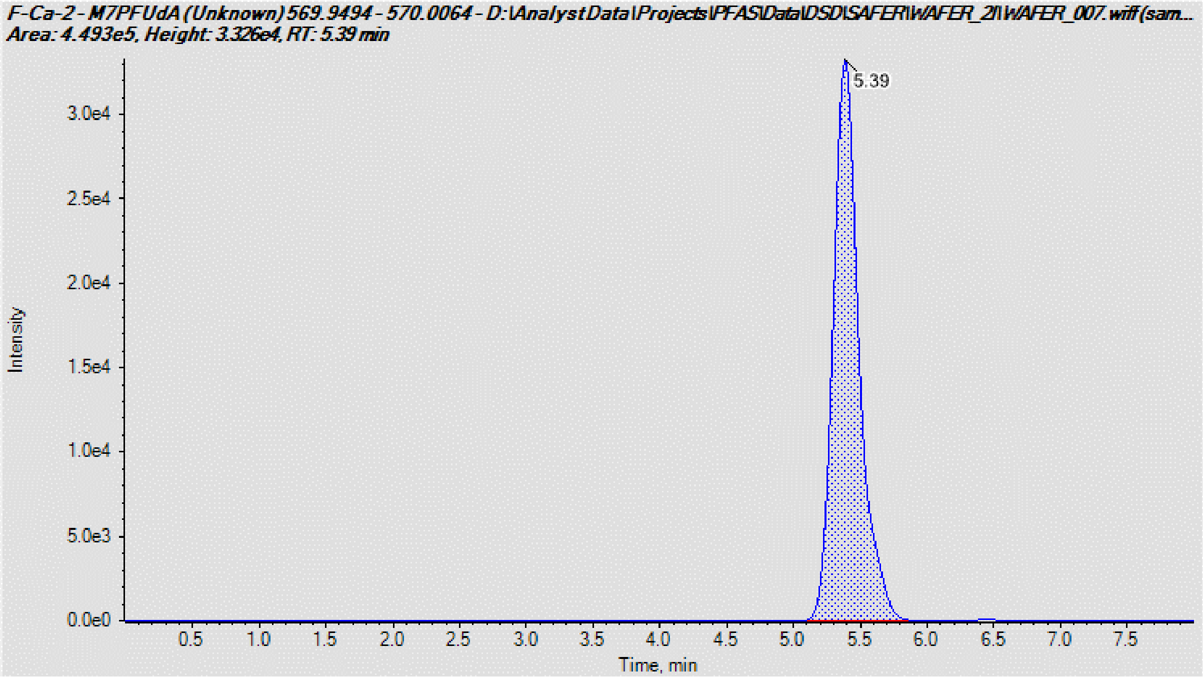
F-Ca-2 - M7PFUdA (Unknown) 569.9494 - 570.0064 - D:\AnalystData\Projects\PFAS\Data\DSD\SAFER\WAFER_2\WAFER_007.wiff (sam...
Area: 4.493e5, Height: 3.326e4, RT: 5.39 min
5.39
Intensity
3.0e4
2.5e4
2.0e4
1.5e4
1.0e4
5.0e3
0.0e0
0.5
1.0
1.5
2.0
2.5
3.0
3.5
4.0
4.5
5.0
5.5
6.0
6.5
7.0
7.5
Time, min

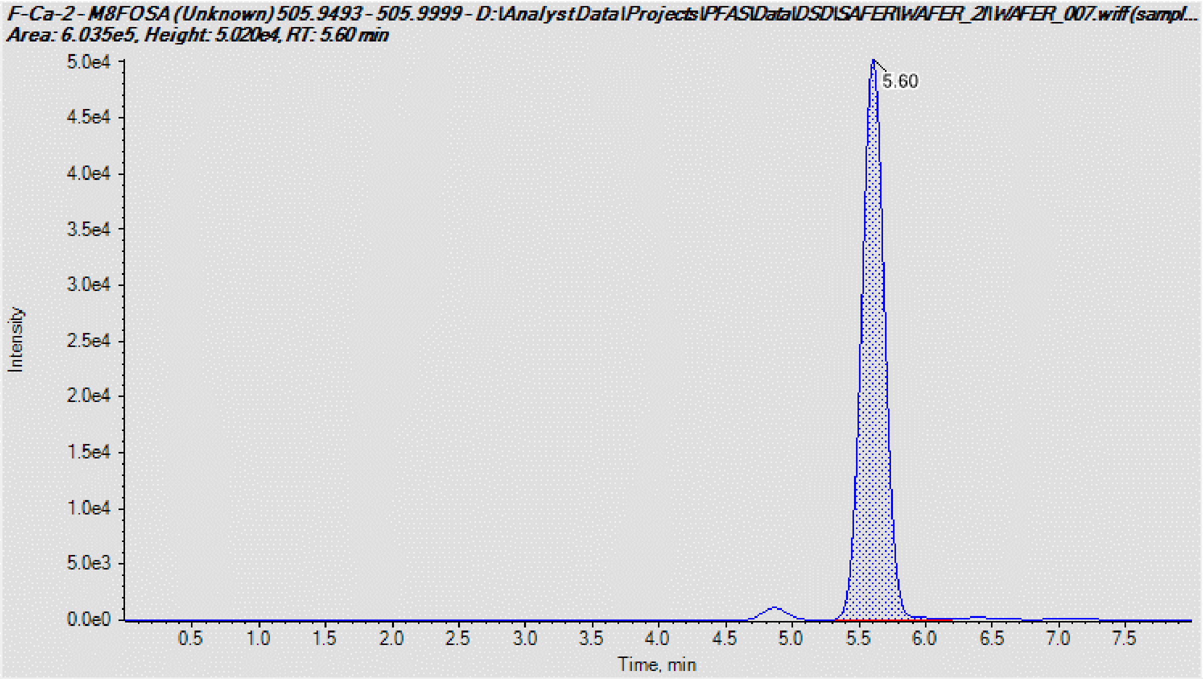
F-Ca-2 - M8FOSA (Unknown) 505.9493 - 505.9999 - D:\AnalystData\Projects\PFAS\Data\DSD\SAFER\WAFER_2\WAFER_007.wiff (sampl...
Area: 6.035e5, Height: 5.020e4, RT: 5.60 min
5.60
Intensity
5.0e4
4.5e4
4.0e4
3.5e4
3.0e4
2.5e4
2.0e4
1.5e4
1.0e4
5.0e3
0.0e0
0.5
1.0
1.5
2.0
2.5
3.0
3.5
4.0
4.5
5.0
5.5
6.0
6.5
7.0
7.5
Time, min

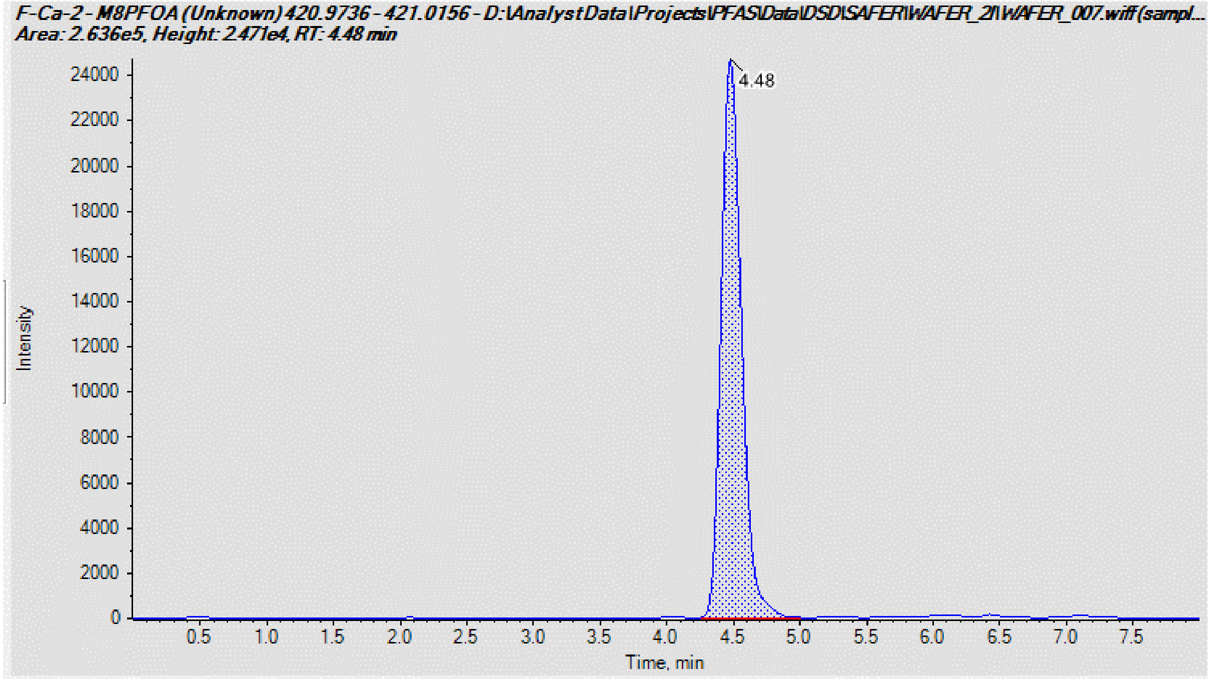
F-Ca-2 - M8PFOA (Unknown) 420.9736 - 421.0156 - D:\AnalystData\Projects\PFAS\Data\DSD\SAFER\WAFER_2\WAFER_007.wiff (sampl...
Area: 2.636e5, Height: 2.471e4, RT: 4.48 min
4.48
Intensity
Time, min

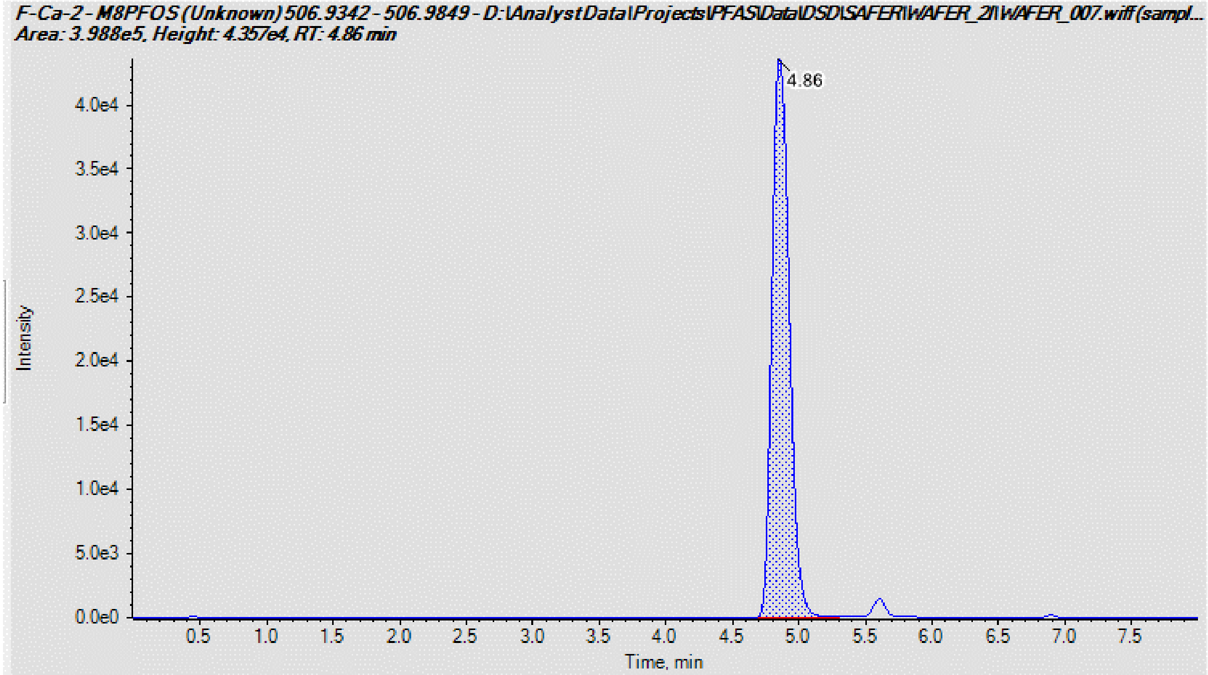
F-Ca-2 - M8PFOS (Unknown) 506.9342 - 506.9849 - D:\AnalystData\Projects\PFAS\Data\DSD\SAFER\WAFER_2\WAFER_007.wiff (sampl...
Area: 3.988e5, Height: 4.357e4, RT: 4.86 min
4.86
Intensity
Time, min

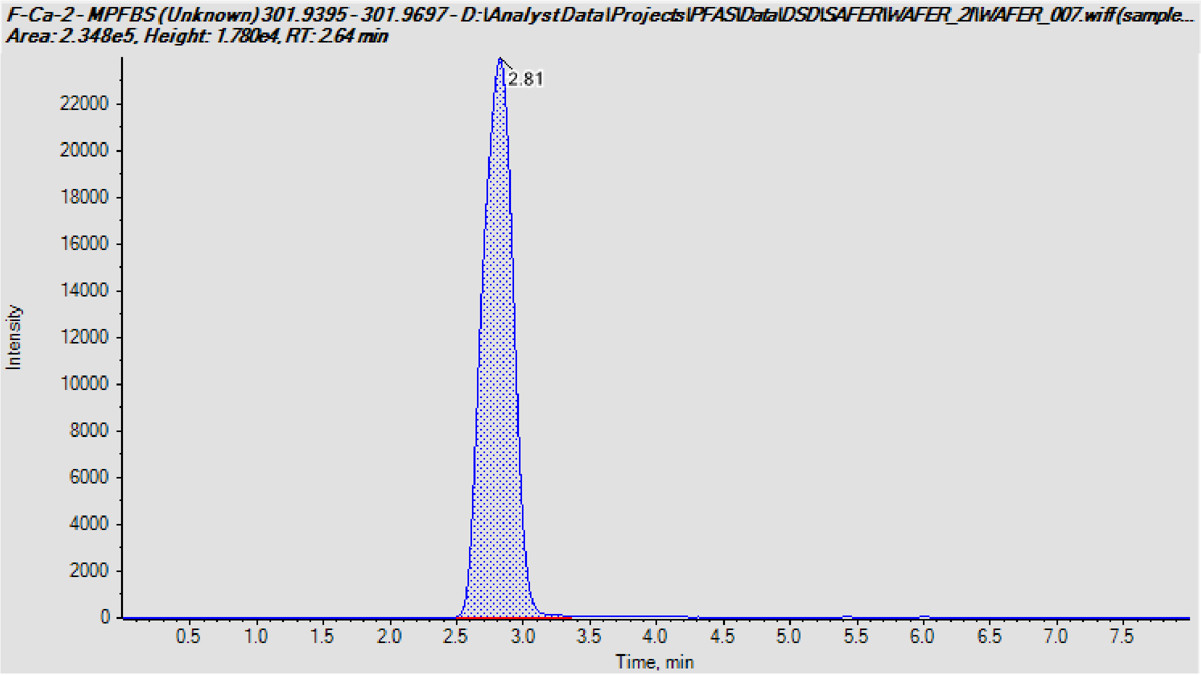
F-Ca-2 - MPFBS (Unknown) 301.9395 - 301.9697 - D:\AnalystData\Projects\PFAS\Data\DSD\SAFER\WAFER_2\WAFER_007.wiff (sample...
Area: 2.348e5, Height: 1.780e4, RT: 2.64 min
2.81
Intensity
22000
20000
18000
16000
14000
12000
10000
8000
6000
4000
2000
0
0.5 1.0 1.5 2.0 2.5 3.0 3.5 4.0 4.5 5.0 5.5 6.0 6.5 7.0 7.5
Time, min

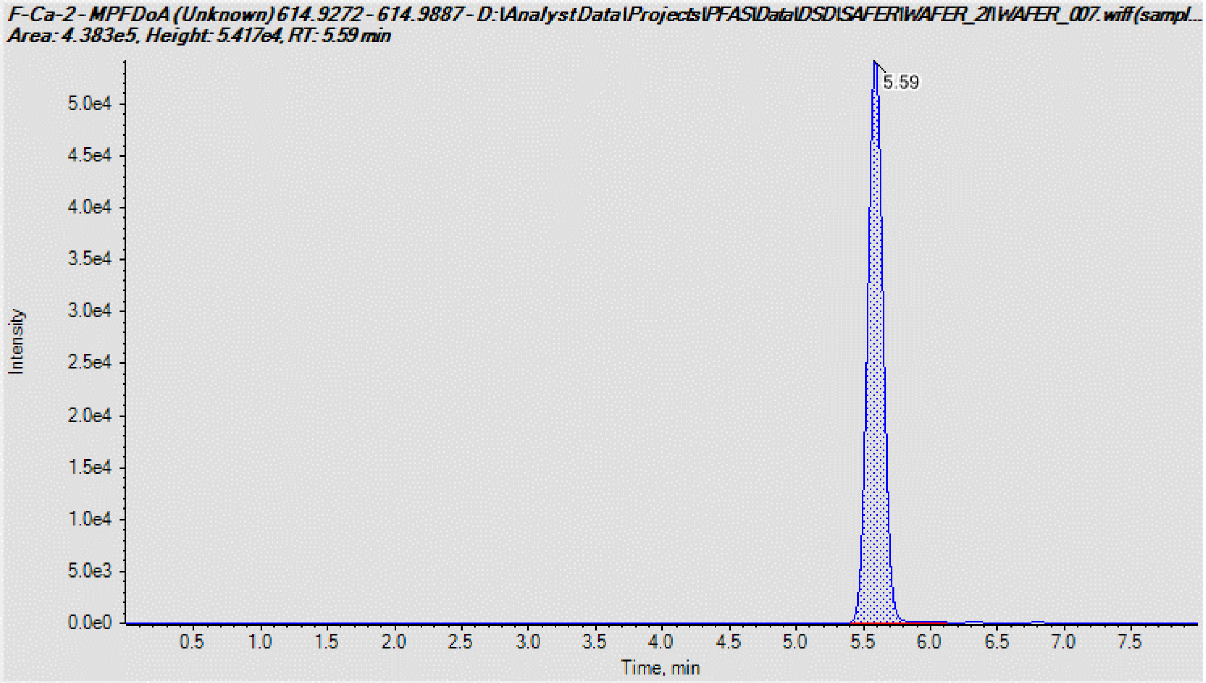
F-Ca-2 - MPFDoA (Unknown) 614.9272 - 614.9887 - D:\AnalystData\Projects\PFAS\Data\DSD\SAFER\WAFER_2\WAFER_007.wiff (sampl...
Area: 4.383e5, Height: 5.417e4, RT: 5.59 min
5.59
Intensity
5.0e4
4.5e4
4.0e4
3.5e4
3.0e4
2.5e4
2.0e4
1.5e4
1.0e4
5.0e3
0.0e0
0.5 1.0 1.5 2.0 2.5 3.0 3.5 4.0 4.5 5.0 5.5 6.0 6.5 7.0 7.5
Time, min

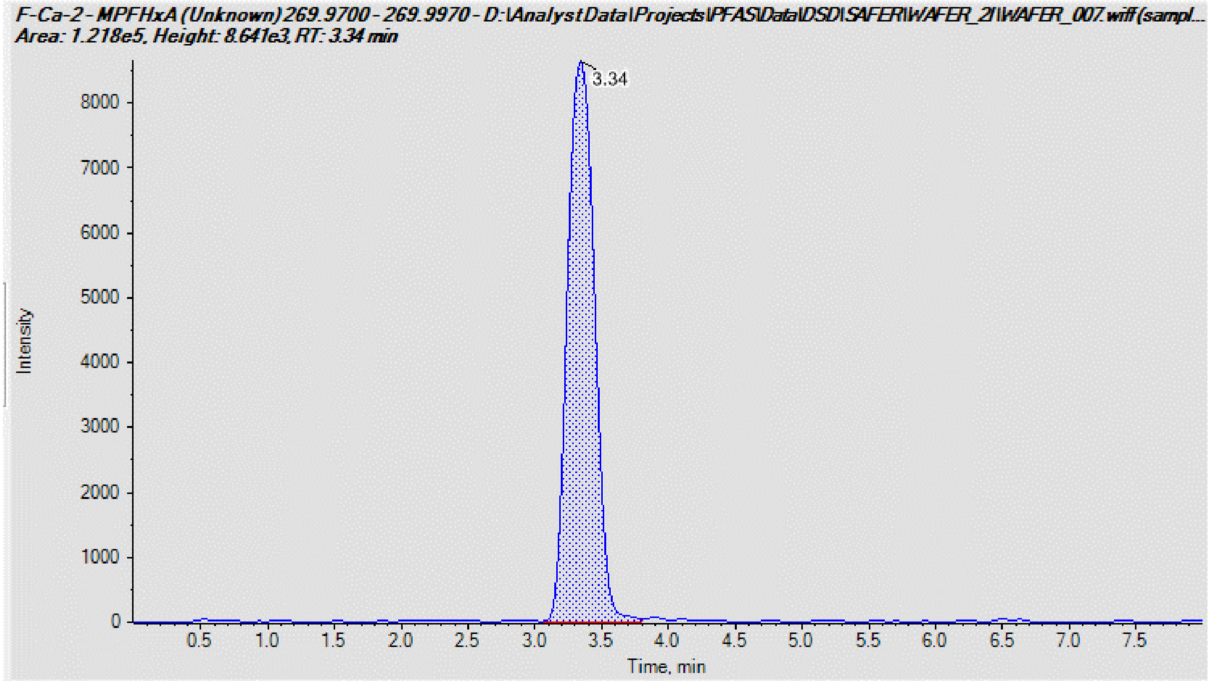
F-Ca-2 - MPFHxA (Unknown) 269.9700 - 269.9970 - D:\AnalystData\Projects\PFAS\Data\DSD\SAFER\WAFER_2\WAFER_007.wiff (sampl...
Area: 1.218e5, Height: 8.641e3, RT: 3.34 min
3.34
Intensity
0
1000
2000
3000
4000
5000
6000
7000
8000
0.5
1.0
1.5
2.0
2.5
3.0
3.5
4.0
4.5
5.0
5.5
6.0
6.5
7.0
7.5
Time, min

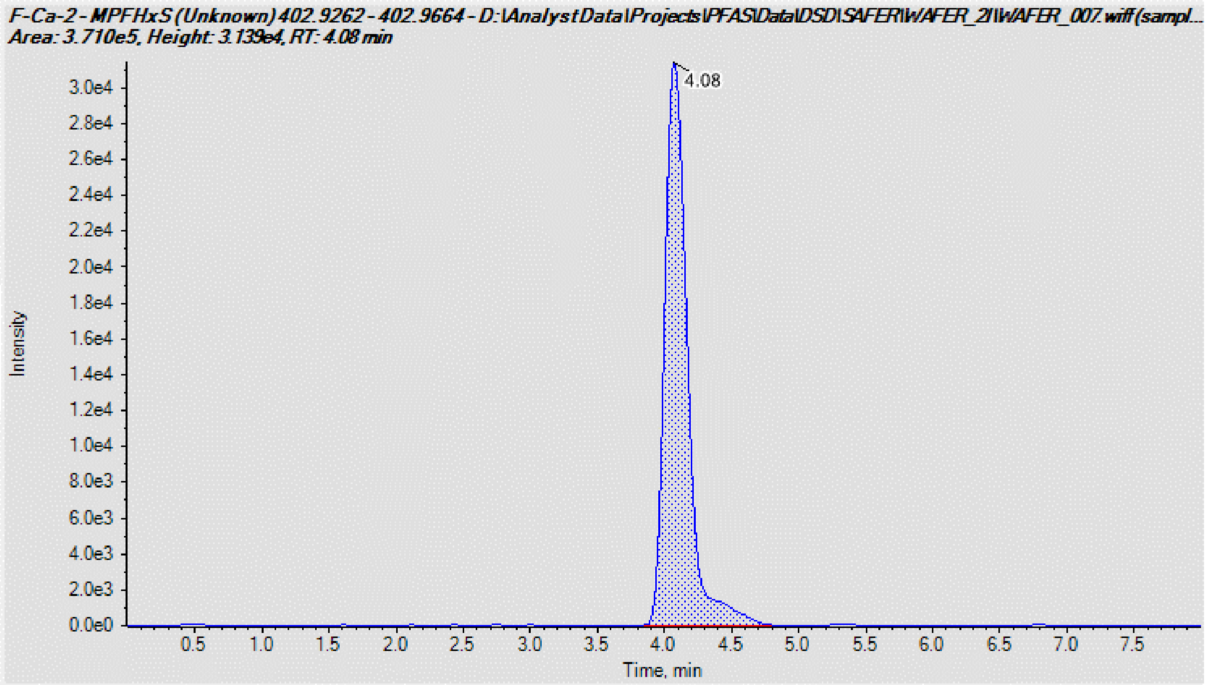
F-Ca-2 - MPFHxS (Unknown) 402.9262 - 402.9664 - D:\AnalystData\Projects\PFAS\Data\DSD\SAFER\WAFER_2\WAFER_007.wiff (sampl...
Area: 3.710e5, Height: 3.139e4, RT: 4.08 min
4.08
Intensity
0.0e0
2.0e3
4.0e3
6.0e3
8.0e3
1.0e4
1.2e4
1.4e4
1.6e4
1.8e4
2.0e4
2.2e4
2.4e4
2.6e4
2.8e4
3.0e4
0.5
1.0
1.5
2.0
2.5
3.0
3.5
4.0
4.5
5.0
5.5
6.0
6.5
7.0
7.5
Time, min

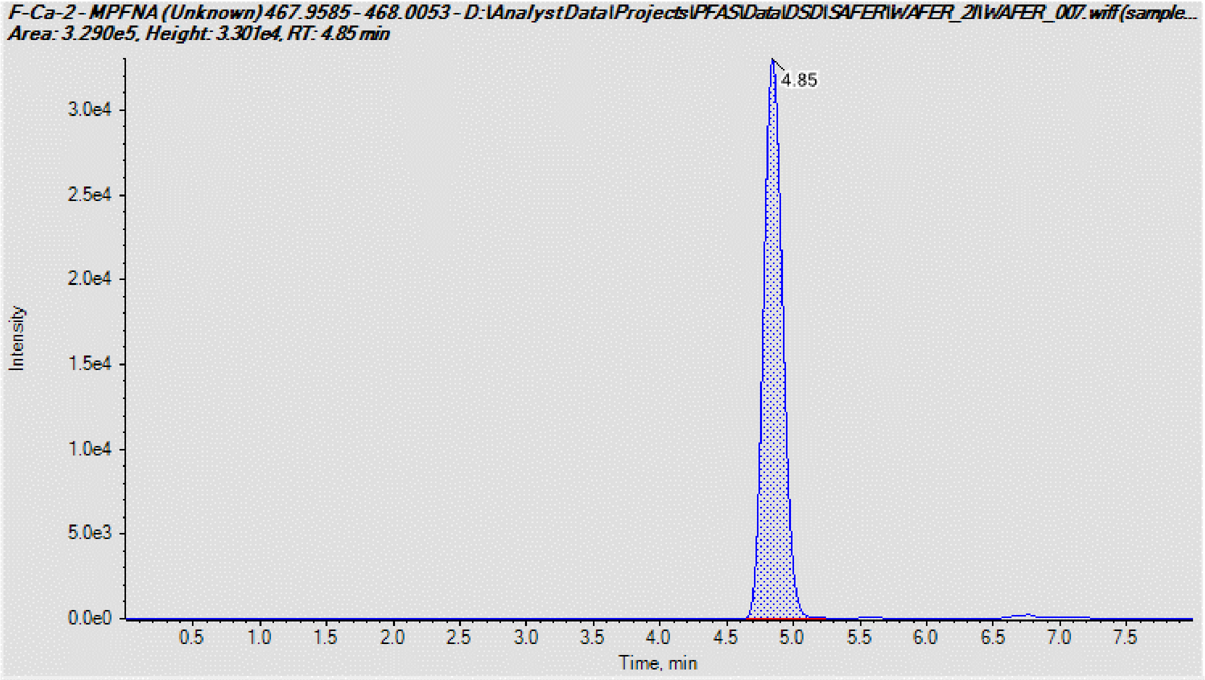

Figure S1. Internal standard chromatograms from a typical sample.
